# Research Status of and Prospects for 3D Printing for Continuous Fiber-Reinforced Thermoplastic Composites

**DOI:** 10.3390/polym15173653

**Published:** 2023-09-04

**Authors:** Yuan Yang, Bo Yang, Zhengping Chang, Jihao Duan, Weihua Chen

**Affiliations:** 1 Key Laboratory of Manufacturing Equipment of Shaanxi Province, Xi’an University of Technology, Xi’an 710048, China; 2 School of Mechanical Engineering, Northwestern Polytechnical University, Xi’an 710072, China

**Keywords:** three-dimensional printing, CFRTPCs, FDM, process parameters, mechanical properties

## Abstract

Continuous fiber-reinforced thermoplastic composites (CFRTPCs) have advantages such as high specific strength, high specific modulus, corrosion resistance, and recyclability and are widely used in the fields of aerospace, rail transit, new energy, and so on. However, traditional methods for preparing CFRTPCs, such as placement and molding, rely more on forming molds, resulting in high manufacturing costs and a slow response speed, which limits the promotion and application of the new generation of CFRTPCs with complex configurations and designable performance. Three-dimensional printing can efficiently create products with multiple materials, complex structures, and integrated functions, introducing new ways and opportunities for the manufacturing of CFRTPCs. However, poor mechanical properties are the bottleneck problem in achieving 3D printing of CFRTPCs. This paper summarizes the research status of the fused deposition modeling (FDM) 3D printing process and the corresponding mechanical properties of CFRTPCs. The focus is on analyzing the influences of the FDM process parameters, such as the material type, printing temperature, speed parameters, layer thickness, scanning space, stacking direction, and fiber volume content, on the mechanical properties of CFRTPCs. Finally, the main problems and future prospects of current CFRTPCs-FDM are analyzed and forecasted, providing new references and ideas for 3D printing of high-performance CFRTPCs.

## 1. Introduction

**Figure 1:**
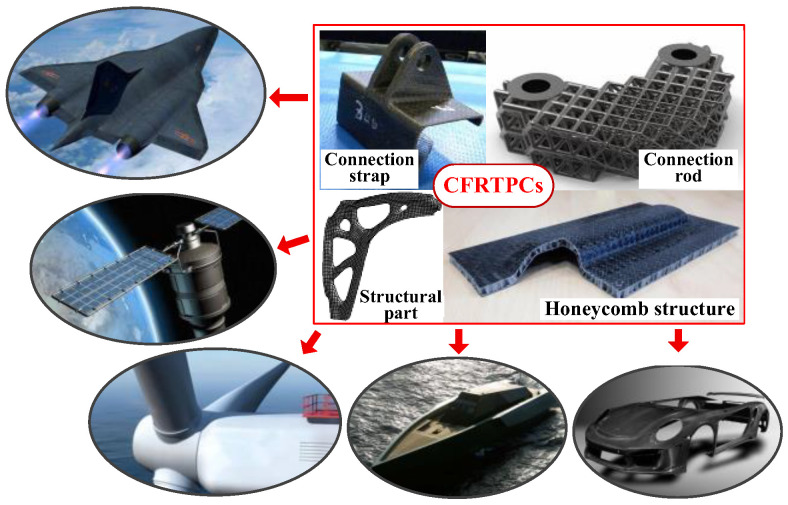
CFRTPCs products and their applications in various fields.

Continuous Fiber-Reinforced Thermoplastic Composites (CFRTPCs), composed of reinforcing fiber and matrix resin through certain forming processes, have advantages such as high specific strength, high specific modulus, corrosion resistance, fatigue resistance, recyclability, good damping and shock absorption, designable performance, and multifunctional integration [1,2,3]. As shown in Figure 1, they have been widely used in various fields such as aviation, aerospace, rail transit, new energy vehicles, nuclear power, wind energy, and so on [4,5].

**Figure 2:**
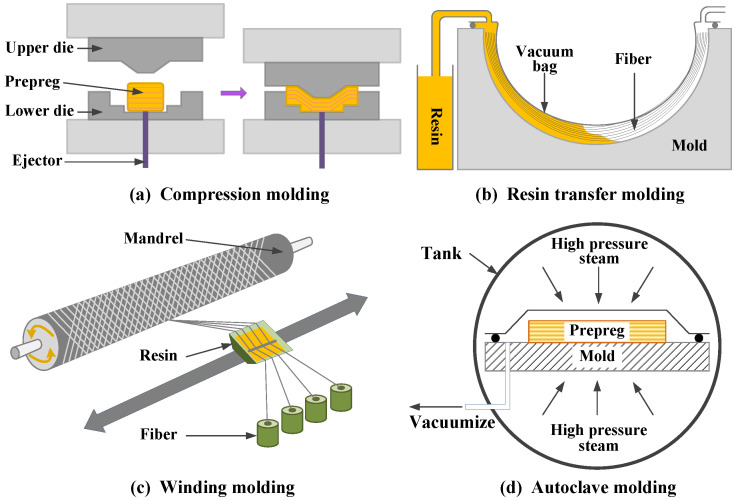
Traditional preparation processes for CFRTPCs.

The traditional process methods for preparing CFRTPCs (such as compression molding [6], resin transfer molding [6], automated winding/placement [7], autoclave molding [8], etc., as shown in Figure 2) are excessively dependent on the forming die, with high manufacturing costs and slow response speeds. However, with the continuous promotion and application of composite materials, the design of the appearance of products is becoming increasingly complex, the accuracy requirement is becoming higher, and the market rapid response and lightweight requirement are becoming more stringent. Relying on traditional process methods, it is difficult to meet the above technical and efficiency requirements of complex high-performance CFRTPCs.

Three-dimensional printing can directly build products with multiple materials, complex structures, and integrated functions, according to the CAD model without the need for traditional mold development and mechanical processing, which makes the forming not limited by the shape of the parts and the mold, and greatly shortens the product development cycle. Therefore, 3D printing of CFRTPCs has become a new generation of composite material forming methods, namely composites 2.0 [4,5,9,10].

Applying 3D printing technology to the field of forming CFRTPCs can fully leverage the manufacturing advantages of 3D printing and the performance advantages of composite materials [11], which can truly achieve the transformation of the design and manufacturing philosophy of CFRTPCs from “design for manufacturing” to “manufacturing for design”. Fused deposition modeling (FDM) is a widely used 3D printing method due to its advantages of low cost, easy operation, and relatively simple process. This paper analyzes and summarizes the current research status of CFRTPCs-FDM. Firstly, the process principles, classification, and corresponding mainstream printing equipment of CFRTPCs-FDM are analyzed and introduced. Secondly, a detailed discussion is conducted on the impact mechanism and research status of the FDM process parameters on the mechanical properties of CFRTPCs. Finally, based on the current research status, the main problems, future research directions, and development trends of CFRTPCs-FDM are considered and prospected.

## 2. CFRTPCs-FDM Principles and Equipment

According to the different ways of embedding continuous fibers into the resin matrix, the process principles of CFRTPCs-FDM can be mainly divided into two categories: online infiltration co-extrusion and offline prepreg double nozzles extrusion.

**Figure 3:**
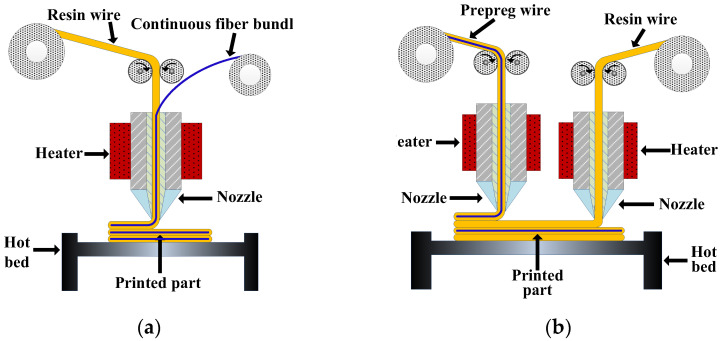
CFRTPCs-FDM principle. (**a**) Online infiltration co-extrusion; (**b**) Offline prepreg double nozzles extrusion.

Online infiltration co-extrusion: As shown in Figure 3a, continuous fiber dry bundles and pure resin wires are used as raw materials, and both are fed into the same printing head. The fibers are embedded in the resin and soaked inside the printing head and then extruded and deposited on the current printing layer together. The infiltrating time and pressure inside the printing head directly affect the infiltrating effect of fibers/resins. By controlling the feeding speed of resin wires, the fiber volume content of the part can be adjusted.

Offline prepreg double nozzles extrusion: As shown in Figure 3b, a prepreg wire premade of continuous fibers and resin is used as the raw material, and the prepreg is melted, extruded, and deposited in one of the printing nozzles. The other printing head uses pure resin wire as the raw material. On the one hand, the pure matrix resin is melted and deposited to form a good bonding interface between the prepreg layers required by the design, and on the other hand, the pure matrix resin can be used to print the outer frame of the product or fill the internal space to obtain a better appearance and dimensional accuracy. By controlling the printing amount of the pure matrix resin, the fiber volume content can be indirectly adjusted.

**Figure 4:**
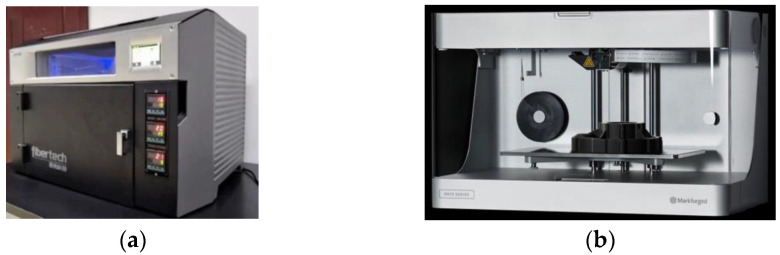
CFRTPCs-FDM printing equipment. (**a**) Combot-1^TM^; (**b**) MarkTwo^TM^.

The representative printer of online infiltration co-extrusion is the Combot series printing equipment developed by Xi’an Jiaotong University, as shown in Figure 4a; the representative printer of offline prepreg double nozzles extrusion is the Mark series printing equipment developed by the MarkForged Company (Waltham, MA, USA), as shown in Figure 4b.

The principle of CFRTPCs-FDM determines the structural form of the 3D printers. For CFRTPCs with the same component, using different process principles and printer structures results in significant differences in the degree of infiltration between the fibers and resins and significant differences in the mechanical properties of the parts. Therefore, when selecting the process principle and corresponding printing equipment, factors such as printing cost, printing efficiency, material performance, and molding quality should be comprehensively considered [12,13].

**Figure 5:**
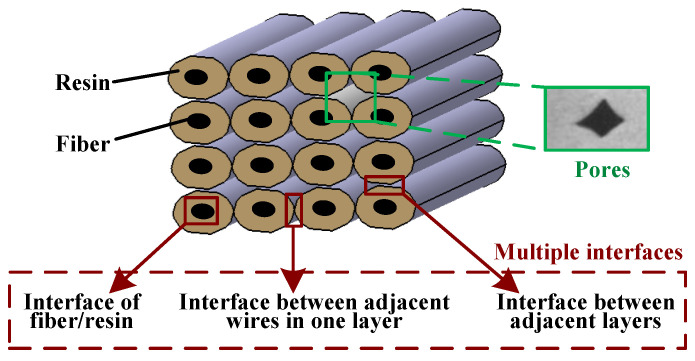
CFRTPCs-FDM printing equipment.

**Figure 6:**
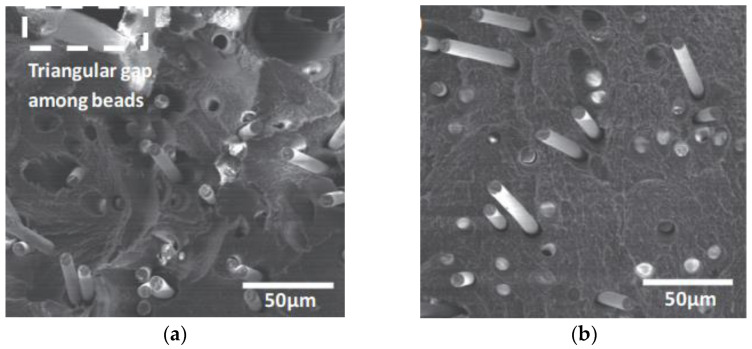
Fracture surface SEM micrographs [15]. (**a**) CF/ABS composites printed by FDM; (**b**) CF/ABS composites formed by compression molding.

At present, CFRTPCs-FDM is still in its early development stages, with problems such as low forming accuracy and poor mechanical properties caused by high porosity and multiple interfaces [14], as shown in Figure 5. Figure 6 shows the fracture surface SEM micrographs of the CF/ABS composites printed by FDM and by compression molding, respectively, indicating that pore enlargement is evident around the fibers in the FDM sample, while no significant enlargement is seen in the compression molding sample. How to efficiently integrate CFRTPCs with AM and achieve rapid and accurate additive manufacturing of CFRTPCs is of great significance.

## 3. Influence of the Process Parameters on the Mechanical Properties

**Figure 7:**
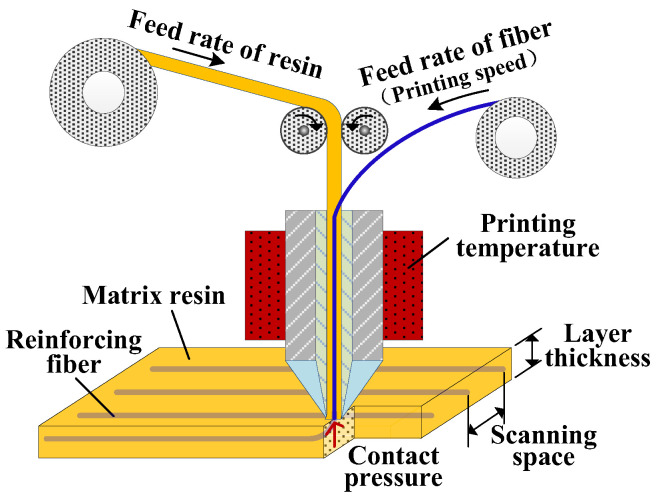
CFRTPCs-FDM based on online infiltration co-extrusion.

The schematic diagram of CFRTPCs-FDM and its process parameters based on online infiltration co-extrusion is shown in Figure 7. The mapping relationship between the process parameters and the mechanical properties is the basis for parameter optimization. Table 1 summarizes the relationship that has been studied in the existing literature, where the mechanical properties include tensile, bending, shear, and compression, and the process parameters include the printing temperature, speed parameters, printing layer thickness, scanning space, etc.

### 3.1. Material Type

CFRTPCs are composed of two types of materials: a matrix phase thermoplastic resin and a reinforcing phase continuous fiber. The continuous fiber is used to enhance the load-bearing capacity, while the resin is used to support and protect the fibers and evenly transmit and distribute loads. Currently, for CFRTPCs-FDM, commonly used fibers include carbon fiber (CF), glass fiber (GF), and Kevlar fiber (KF); commonly used thermoplastic resins include polylactic acid (PLA), acrylonitrile butadiene styrene copolymers (ABS), polyamide (commonly known as nylon) (PA), and polyether ether ketone (PEEK). Table 2 shows the mechanical parameters of the materials.

Different fiber/resin combinations result in significant differences in the mechanical properties of the 3D printed CFRTPCs due to the different interphase interface properties. Oztan et al. [16] used CF and KF to enhance the nylon matrix and found that the tensile strength of the 3D printed part increased by 2 to 11 times, reaching the strength level of aviation aluminum alloys. In the literature [17,18,19,22], CFRTPCs were 3D printed with continuous CF, GF, and KF as reinforcing phases and nylon as the matrix phase, respectively. It was found that the mechanical properties of the CF-reinforced composites were the best, followed by GF, and KF was the worst. In addition to the differences in the properties of the fibers themselves, Dickson et al. [17], Caminero et al. [21], and Goh et al. [20,31] also found in the study of the interlayer mechanical properties that the interfacial bonding performance between the reinforced fibers and the matrix is an important factor affecting the mechanical properties of the workpiece.

The above research indicates that the material properties of fibers and resins, as well as their interfacial properties, are important factors affecting the mechanical properties of CFRTPCs. To improve the mechanical properties, on the one hand, efforts can be made to develop high-performance fibers and matrices and expand the material system; on the other hand, fiber modification technology can be combined to make the fiber surface rougher or undergo chemical reactions to generate new polar groups to promote matrix impregnation of the fibers and improve the interfacial bonding performance between the fibers and the matrix.

### 3.2. Printing Temperature

The printing temperature directly affects the melt flow performance of the matrix resin after heating, affecting the mechanical properties of the CFRTPCs from two aspects:

(1) Influence on the infiltration between fibers and resins

The printing temperature is too low, resulting in high viscosity and poor fluidity during resin melting, making it difficult to complete the printing work. The increase in printing temperature enhances the resin melt fluidity, making it easier to enter the interior of the fiber bundle, improving the degree of infiltration between the fibers and resin, and thus improving the forming quality of the workpiece. However, excessive temperature can easily lead to strong resin fluidity and even decomposition and vaporization, which is not conducive to 3D forming. In general, the reasonable range for 3D printing temperature selection is above the glass transition temperature of the resin material and below the thermal decomposition temperature. Tian et al. [27] confirmed this through experiments. When the printing temperature is below 180 °C, the fluidity of the PLA is poor, and the nozzle is prone to blockage. When the temperature is above 240 °C, PLA is in liquid form and naturally flows out from the nozzle, which cannot guarantee printing accuracy. Based on comprehensive analysis, it can be concluded that the reasonable printing temperature for PLA is 180–230 °C. Shan et al. [25] found that when the printing temperature increased from 180 °C to 220 °C, the tensile strength of the specimen increased by about 20% (188 MPa vs. 225 MPa), and the bending strength increased by about 8% (274 MPa vs. 296 MPa).

(2) Influence on the multi-interface bonding performance of CFRTPCs

Three-dimensional printed CFRTPCs have multiple interfaces, as shown in Figure 6, mainly including the interphase interface of fiber/resin, the interface between adjacent layers, and the interface between adjacent wires in the layer, among which the latter two belong to the resin wire interface.

**Figure 8:**
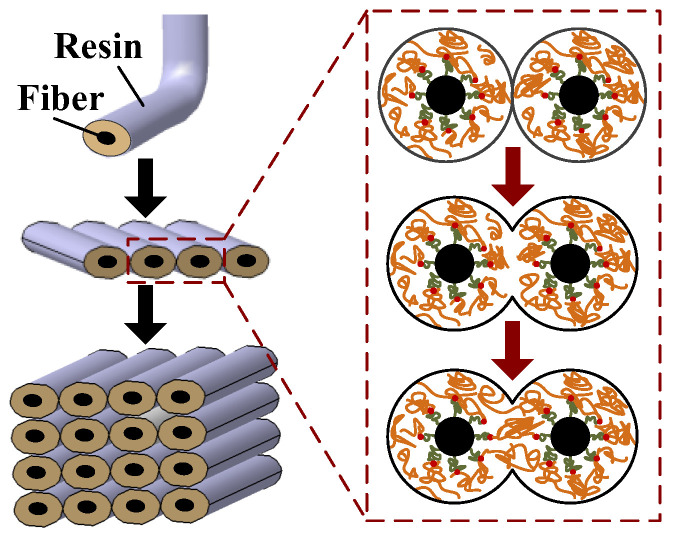
Multi-interface formation process of CFRTPCs-FDM.

(1) For the fiber/resin interface, a reasonable printing temperature is beneficial for the resin matrix to remain above the glass transition temperature for a relatively long time, which is conducive to the full infiltration between the resin and the fiber bundle, thereby obtaining a phase interface with good performance.
(2) For the interface between resin wires, as shown in Figure 8, the formation process of the wire interface includes three stages: the contact between the wire surfaces, the radial growth between adjacent wires, and the diffusion and fusion of molecular chains. A reasonable printing temperature is conducive to the full diffusion and fusion of molecular chains near the contact surface of the printed wire, promoting the radial growth of the wire contact surface, and finally forming a good wire interface.

### 3.3. Speed Parameters

The speed parameters include the printing speed and substrate feeding speed. The printing speed refers to the movement speed of the printing head, which is also the wire output speed of continuous fibers at the printer nozzle. The printing speed determines the forming efficiency of the workpiece and the soaking time of the continuous fibers and molten resin in the printing head. For online infiltration co-extrusion, the substrate feeding speed refers to the volume of resin material entering the printing head per unit time. Since the nozzle inner diameter remains unchanged, the substrate feeding speed determines the impregnation pressure between the fiber and the molten matrix, and to some extent determines the fiber volume content of the product.

When determining the speed parameters, it is necessary to comprehensively consider the nozzle diameter and the type and performance of the fiber and resin. In general, a lower printing speed and a higher substrate feeding speed can improve the impregnation pressure and degree, which is beneficial for improving the interfaces’ bonding performance. However, too low a printing speed can prolong the heating time of the resin matrix inside the nozzle, causing thermal decomposition of the resin and preventing normal printing. Excessive substrate feeding speed may cause the molten resin to overflow from the nozzle, affecting the normal printing work. Zeng et al. [28] also found that the bending strength and modulus of the specimen decreased with the increasing printing speed (1 mm–6 mm/s). Dou et al. [23] concluded that the tensile strength and modulus of the CF-reinforced PLA specimens decreased by 7.70% (200.43 MPa vs. 185 MPa) and 17.07% (23.31 GPa vs. 19.33 GPa), respectively, by increasing the printing speed from 50 mm/min to 400 mm/min.

### 3.4. Layer Thickness

As shown in Figure 5, the printing layer thickness refers to the spatial distance between the nozzle and the previous printing layer. A smaller layer thickness is beneficial for enhancing the compaction effect of the nozzle on the material during the process [37,44,45]. On the one hand, it can promote the resin in the molten state to better infiltrate the reinforced fibers and improve the fiber/matrix interface performance. On the other hand, it is also beneficial for improving the interface performance between adjacent printing wires within and between layers. At the same time, the layer thickness also affects the fiber volume content and porosity in the material. As the thickness decreases, the fiber volume content of the product shows an upward trend, and the corresponding porosity decreases.

For 3D printed CFRTPCs, Shan et al. [25] found that with the increase in the layer thickness (0.8–1.2 mm), the tensile strength and bending strength of the workpiece decreased by 33.82% (253.28 MPa vs. 167.63 MPa) and 37.17% (224.78 MPa vs. 141.22 MPa), respectively. Tian et al. [27] found that with the increase in the thickness (0.3–0.8 mm), the bending strength and modulus decreased by 58.9% and 66.3%, respectively. Hu et al. [29] found in their study that the thickness has the most significant impact on the bending performance of composite materials. As the thickness decreases, the bending strength and modulus of the 3D printed continuous CF-reinforced PLA composite can reach 610.1 MPa and 40.1 GPa, respectively. The reduction in the pore defects and the increase in the fiber volume content in materials are the main reasons for the significant improvement in the bending performance. Ning et al. [24] also found that a decrease in the thickness can reduce the porosity of the composite, thereby improving the mechanical properties. Chacón et al. [18,21,22,46] found in 3D printing research that an increase in the layer thickness leads to a decrease in the printing efficiency, and the impact of the thickness on the material mechanical properties is also related to the specific material stacking direction and load action form. Ming et al. [33] found that when the printing layer thickness is large, weak compaction leads to poor interface bonding performance between adjacent layers. But when the thickness is too small, the strong compaction effect can lead to fiber breakage and printing nozzle blockage. Only when the thickness is reasonably selected, can the surface of composite materials be relatively flat, the material can have good fiber continuity and low porosity, and the mechanical properties of the composite are at their highest.

Therefore, when selecting the thickness of the printing layer, it is necessary to comprehensively consider the relationship between the material mechanical properties, printing accuracy, and printing efficiency and make a compromise.

### 3.5. Scanning Space

As shown in Figure 5, the scanning space refers to the center distance between adjacent printing wires within the same printing layer. Usually, to ensure sufficient contact between printed wires and reduce porosity, a certain overlap area is required. Different scanning spaces can lead to differences in overlap and contact pressure, which in turn affect the degree of fiber/resin infiltration and the multi-interface bonding performance. When the scanning space is too small and the overlapping proportion is too high, fiber wear and fracture occur in the printed structure. If the scanning spacing is too large, there is no overlap between the adjacent wires, and there are obvious pore defects. And the different scanning space also affects the fiber volume content and mechanical properties of the composites.

In the study of the 3D printing of CFRTPCs, Shan. et al. [25] found that with the increase in the scanning space (0.5–1.1 mm), the tensile strength and bending strength of the composites increased first and then decreased. When the scanning space was 0.65 mm, the mechanical property was the highest. When the scanning space was less than 0.65 mm, fiber wear, fracture and warpage occurred during printing, resulting in poor mechanical properties. When the space was greater than 0.65 mm, the increase in the scanning space reduced the impregnation degree and also caused the decrease in the fiber volume content, which led to the decrease in the mechanical strength. Tian et. al. [27,38] discussed the effect of the scanning space on the flexural properties in detail. It was found that with the increase in the scanning space (0.4–1.8 mm), the flexural strength and flexural modulus decreased by 60.7% and 79.3%, respectively. In summary, the scanning space has a significant effect on the mechanical properties of the composites.

### 3.6. Stacking Direction

**Figure 9:**
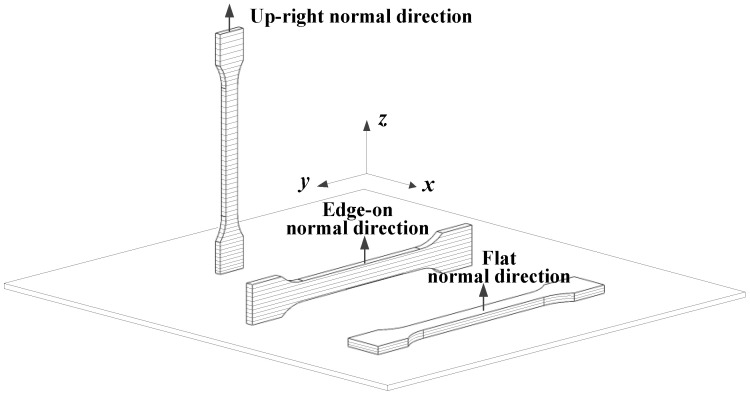
Schematic diagram of stacking direction.

According to the different geometric shapes and performance requirements of the parts, different stacking directions can be used to stack and accumulate materials layer by layer. The stacking direction is also an important factor affecting the mechanical properties of CFRTPCs. As shown in Figure 9, in the x-y-z coordinate system, the *z*-axis represents the stacking direction. It can be seen that the standard test pieces with the same geometric shape have various stacking directions such as a flat normal direction, a side legislative direction, and a straight legislative direction. Three-dimensional prints with different stacking directions have different microstructures and mechanical properties.

Currently, most research on the stacking direction is mainly based on experiments. Chacón et. al. [18,22,46] systematically studied the influence of the three stacking directions shown in Figure 7 on the mechanical properties of CFRTPCs. In terms of the tensile tests, the performance of stacking along the flat normal and side legislative directions was close to and good, while the performance of stacking along the straight legislative direction was poor. In terms of the bending and impact experiments, the performance of stacking along the lateral direction was the best, followed by the horizontal normal direction and the vertical normal direction. It is worth noting that the impact of the stacking methods on the mechanical properties varies under different loads [41]. The reasons can be summarized: (a) the microstructure of composites with different stacking directions is different, resulting in different bearing characteristics under different loads; (b) different stacking directions can also affect the fiber volume content; (c) different stacking directions may also lead to differences in the porosity of the composite materials.

### 3.7. Fiber Volume Content

Reinforcing fibers are the main load-bearing phase of CFRTPCs, and their volume content directly determines the part’s mechanical properties. Normally, an increase in fiber volume content results in a significant improvement in the mechanical properties. The commonly used methods for calculating fiber content include thermogravimetric analysis [16], geometric calculation [23,37], and image analysis [20,39]. At present, the fiber volume content of 3D printed CFRTPCs does not exceed 50% [11,17,19,47,48], which is lower than that of composite materials prepared by traditional processes. The low fiber volume content is one of the important reasons for the low mechanical properties of 3D printed CFRTPCs. Since in the 3D printing process, with the increase in the fiber volume content, the full infiltration of fibers becomes more difficult, this results in a decrease in the interfacial bonding strength between the fiber/resin phases of 3D printed composites. At the same time, it also introduces more pore defects in the material, ultimately having an adverse impact on the mechanical properties of the composite. Dickson et. al. [17] found that the tensile properties of 3D printed composite materials significantly improved with the increase in the fiber volume content within a certain range. However, when the fiber volume content exceeded a certain level, the improvement in the mechanical properties of the composite material decreased. Chacón et. al. [18,22] believe that an increase in fiber volume content will have two distinct effects on 3D printed composite materials: on the one hand, an increase in the fiber content will hinder the damage evolution in composite materials and play a positive role in improving the mechanical properties of the material; on the other hand, an increase in fiber content will make it more difficult for the fibers to fully infiltrate, leading to a decrease in the strength of the fiber/matrix interface and the introduction of more pore defects in the material, ultimately affecting the mechanical properties. It should be noted that the fiber volume content of 3D printed composite materials is still relatively low now, and with the increase in the fiber volume content, the sufficient infiltration of fibers cannot be effectively guaranteed, and the number of pore defects in the material also increases, which has adverse effects on the improvement of the mechanical properties of composite materials.

Therefore, in future research, on the one hand, effective methods to increase the fiber volume content of 3D printed composite materials should be further explored; on the other hand, in-depth research should also be conducted on how to improve the sufficient infiltration and high porosity of fibers under high fiber volume content, in order to ultimately achieve effective printing of high fiber volume content composite materials with good mechanical properties, meeting the application requirements of complex engineering structures for high-performance composite materials.

## 4. Improvement and Perfection for CFRTPCs-FDM

### 4.1. Structural Topology Optimization and Fiber Path Planning for CFRTPCs-FDM

**Figure 10:**
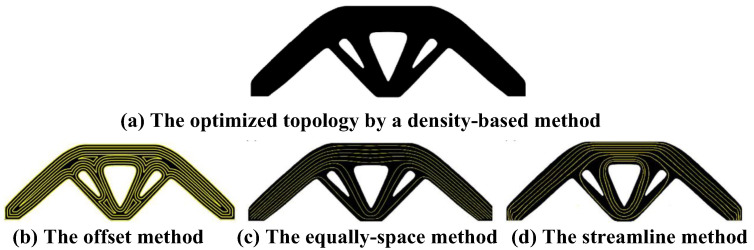
Stiffness-based optimization for the topology and fiber paths by Papapetrou.

Structural topology optimization is an important means to achieve lightweight in CFRTPCs-FDM products, and fiber path planning aims to determine the distribution of continuous fibers within the product. The main challenge of CFRTPCs-FDM structural topology optimization and fiber path planning is the coupling between structural design and the fiber distribution. Due to the anisotropic mechanical properties of fibers, the change in the fiber distribution caused by the shape change of composite materials should be fully considered in the topology optimization process. There are two main ideas for topology optimization and path planning of a CFRTPCs-FDM structure. One is sequential optimization, which first considers the overall topological shape and then considers the fibers distribution. For example, Li et al. [49] and Fedulov et al. [50] proposed a method of additive manufacturing of continuous carbon-fiber-reinforced nylon composites based on path planning. The topological optimization method is used to analyze the transmission path of the load in isotropic materials, and then the fiber trajectory design is carried out. This method considers the load transmission path of continuous fibers and the anisotropic mechanical properties. Papapetrou et al. [51] proposed a shape optimization method based on the density shape level set (Figure 10a) and gave three fiber filling methods: the offset method (Figure 10b), the equally spaced method (Figure 10c), and the streamline method (Figure 10d). These three methods can ensure the continuity of the fiber in the filling area.

The other is a parallel optimization that considers both the structural design and fiber distribution. For example, Lee et al. [52,53] proposed a topology optimization method for functionally graded composite structures, which can simultaneously design the optimal composite topology and spatially variable fiber distribution. A three-dimensional topology optimization method of continuous fibers based on the natural evolution method proposed by Alexander [54] can dynamically find the local optimal distribution of the material density and obtain a lighter structure. Huang et al. [55] proposed a design and manufacturing strategy integrating concurrent optimization of fiber orientation and structural topology for CFRTPCs, realized by ingenious path planning for the 3D printing process.

### 4.2. Assisted Processes and Devices for CFRTPCs FDM

(1) Microwave-heating-assisted CFRTPCs-FDM

**Figure 11:**
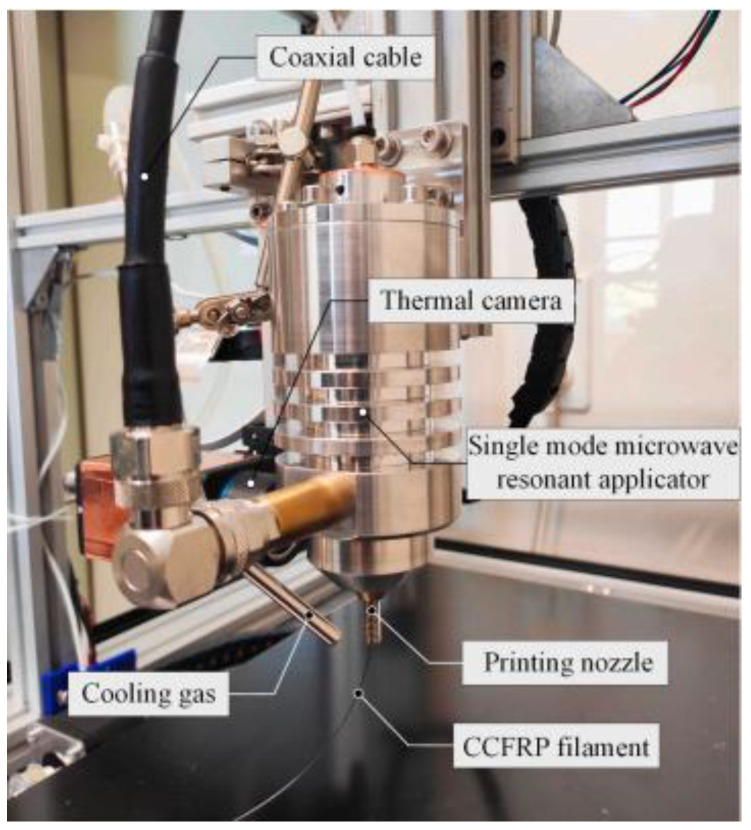
Illustration of the microwave printing head.

At present, the 3D printing of composite materials mainly adopts the traditional electric heating method to melt and then solidify and deposit, which has a long curing time and a high energy consumption. Li et al. [56] applied microwave technology to the 3D printing of composite materials and proposed a microwave-assisted heating CFRTPCs-FDM method, which uses microwaves to instantaneously volume heat CFRTPCs filaments. As shown in Figure 11, microwaves can quickly heat the fiber and quickly transfer heat to the resin, reducing the temperature change caused by the change in the printing speed, thereby improving the mechanical properties of the parts. At the same time, compared with the traditional heating method, the maximum printable wire diameter (1.75 mm vs. 5.48 mm) and the maximum printable speed (10 mm/s vs. 35 mm/s) are increased. Microwave-assisted CFRTPCs-FDM has the advantages of a fast heating speed, high thermal energy utilization, easy control, energy savings, and environmental protection.

(2) Ultrasonic-assisted CFRTPCs-FDM

**Figure 12:**
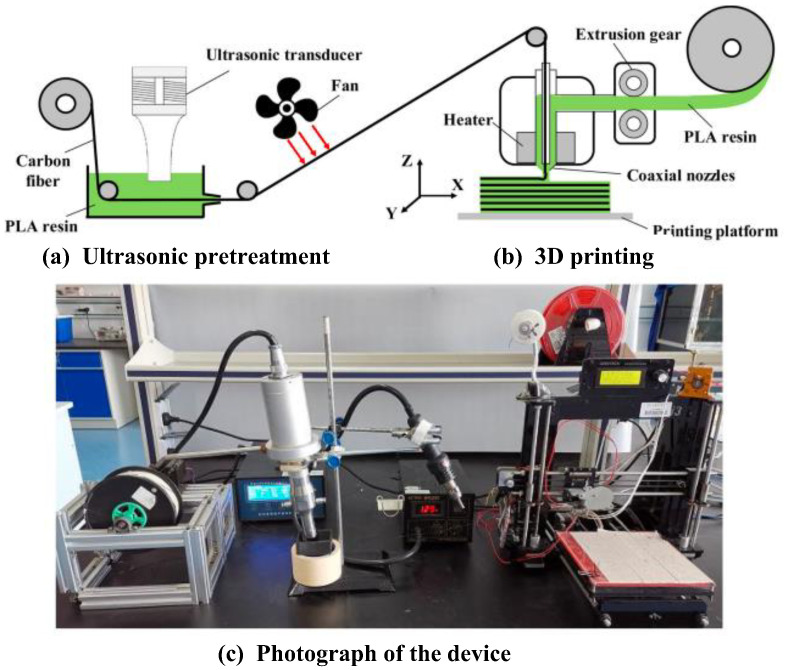
A schematic diagram and a photograph of the ultrasonic-assisted CFRTPCs-FDM.

Qiao et al. [57] proposed an ultrasonic-assisted fiber interface modification method to improve the wettability of fiber resin and the mechanical properties of CFRTPCs parts. In the 3D printing process of carbon fiber composites based on online infiltration co-extrusion, the carbon fiber is first guided to infiltrate once in the resin liquid with ultrasonic action, which reduces the porosity and improves the interfacial properties of the composites, as shown in Figure 12.

In order to improve the bonding between the resin and the fiber and reduce the porosity to improve the printing quality of CFRTPCs, in addition to the above process improvements, scholars have also proposed assisted measures such as substrate ultrasonic vibration assistance, laser melting assistance, roller pressure followup assistance, and hot pressing post treatment to improve the mechanical properties of CFRTPCs–FDM.

### 4.3. Recycling and Remanufacturing of CFRTPCs–FDM

With the wide application of CFRTPCs-FDM, the recycling and remanufacturing of waste composite parts has become a problem that must be faced. The recovery technologies of carbon fiber composite materials are divided into three categories: the physical recovery method, the energy recovery method, and the chemical recovery method. The physical recovery method is to mill, cut, or crush the waste composite materials to obtain short fibers, particles, powders, and other substances. This method has the advantages of being low cost, a simple process, and having no pollution, which is only suitable for uncontaminated composite waste parts, and the strength of the fiber is seriously reduced after treatment, and the reuse value is low. So, it can only be used as some fillers and cannot become a material with high purity requirements. The energy recovery method is to incinerate waste composite materials to obtain heat energy. However, this method causes secondary pollution, which should be avoided as much as possible. The technology that can really further recycle and reuse fibers is the chemical recovery method. The method is to degrade the resin matrix into small molecular compounds or oligomers, so as to achieve separation from fibers and fillers. It can not only recover the reinforced fibers but also recover the resin as raw material. It is the most promising recovery method at present [58].

**Figure 13:**
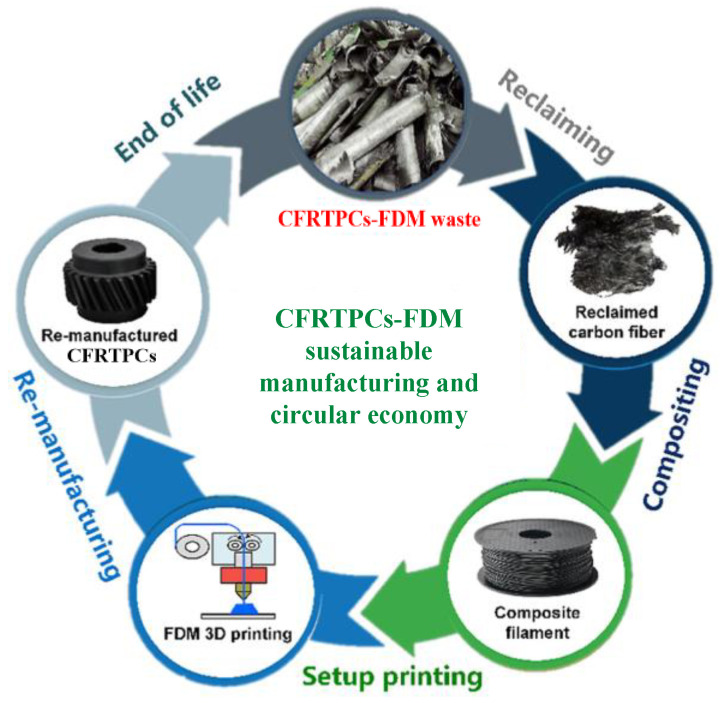
CFRTPCs-FDM sustainable manufacturing and circular economy.

The carbon fiber obtained by the chemical recovery method is usually in a messy and fluffy state, which is difficult to make into prepreg products with long tow lengths. Therefore, the recovered fiber is more suitable for nonwoven materials, cut into short fibers, or even ground into smaller particles or powders, added to the resin matrix for remanufacturing. At present, chopped-fiber-reinforced thermoplastic materials are widely used in the 3D printing of composite materials. Therefore, the recycled carbon fiber is particularly suitable for the 3D printing of chopped-fiber-reinforced composites. Figure 13 shows the CFRTPCs-FDM sustainable manufacturing and circular economy [59]. Compared with original fibers, using recycled fibers for 3D printing composite materials not only avoids the environmental pollution caused by waste materials but also helps to reduce the cost of the 3D printing composite materials.

## 5. Summary and Prospects

Compared with traditional preparation processes, the mechanical properties of 3D printed CFRTPCs are still relatively poor. This paper analyzes and reviews the current research status of the 3D printing of CFRTPCs from the perspective of mechanical properties, and draws the following conclusions:

(1) The mechanical properties of 3D printed CFRTPCs are closely related to the properties of reinforcing fibers, matrix resins, and multiple interfaces. The influencing factors can be summarized into three aspects: the material type, the process principle, and the process parameters. The factors are coupled with each other, jointly determining the forming quality of CFRTPC products;
(2) The impact of the above factors on the mechanical properties of 3D printed CFRTPCs is ultimately reflected in three aspects: the fiber volume content, the interface bonding strength, and the porosity. Compared with traditional forming processes, current 3D printed CFRTPCs still face prominent problems such as a low fiber volume content, a weak interface bonding strength, and a high porosity.
(3) The fiber volume content is determined by a combination of speed parameters, layer thickness, scanning space, stacking direction, and so on. The pores of workpieces can be divided into macroscopic pores caused by the minimum bending radius or scanning overlap of the fibers and microscopic pores existing in continuous fibers, matrix resin, and interphase interface connections. Three-dimensional printed CFRTPCs have multiple interfaces: a fiber/resin interface and a resin interface between and within layers. Good interface bonding characteristics are conducive to reducing the porosity, improving the stress transfer efficiency, and interlaminar shear strength at the interface.

However, the existing research mainly focuses on analyzing the relevant factors through experimental methods along the lines of equipment development, experimental design, and result comparison, and exploring the optimal process methods and parameters under the relevant material types. In the future, research on CFRTPCs-FDM may focus on the following aspects:

(1) It is necessary to explore the rheological and time-dependent behavior of composite materials in the multiple states of FDM from a more microscopic perspective, so as to construct a mechanical model that accurately describes the complex rheological properties of materials and reveal the melting deposition mechanism of CFRTPCs at the molecular/atomic level, to realize scientific prediction of the mechanical properties of 3D printed CFRTPCs.
(2) The data show that 50% to 60% of the structural failures in composite materials are closely related to interlayer damage. The bonding ability of the CFRTPCs’ 3D printing multi-interface (interphase interface, interlayer, and inner layer wire interface) determines the interlaminar mechanical properties of the CFRTPCs to a large extent. Therefore, it is necessary to further study the interface cross scale coupling model of CFRTPCs-FDM, in order to reveal the regulatory mechanism of the interface from the microstructure to the macroscopic performance.
(3) Researchers have established a relatively effective numerical simulation model for the traditional forming process of composite materials, achieving prediction and simulation of their mechanical behavior under typical load conditions. However, numerical simulations for the 3D printing of CFRTPCs are relatively lacking. Effective finite element and molecular dynamic models should be established based on the rheological mechanism and interface model of CFRTPCs-FDM to achieve effective simulation and prediction of 3D printed CFRTPCs properties.
(4) The 3D printing of CFRTPCs has anisotropic characteristics, and the stacking direction and fiber orientation seriously affect the optimal load-bearing condition of the product. Therefore, it is necessary to carry out configuration design in combination with topology optimization, develop five-axis 3D printing equipment, adjust the stacking direction in real time, and use a path planning algorithm to realize the controllable layout of the fiber direction, so as to realize the integrated design and manufacturing of CFRTPCs in terms of “performance–configuration–process”.
(5) It is necessary to explore and develop new process principles and improvement methods for the 3D printing of CFRTPCs, innovate CFRTPCs’ 3D printing equipment, and further improve the mechanical properties. Also needed is to develop new materials and improve the material system and, on the basis of the tensile, bending, and compression properties, further enrich the quality evaluation methods of the 3D printing of CFRTPCs, such as the impact properties, wear properties, creep properties, fatigue properties, and damage evolution laws.

## Figures and Tables

**Table 1:** A list of the literature on the parameters and performance relationship of CFRTPCs.

	Tensile/Elastic	Bending/Flexural	Shear	Compressive	Impact	Fracture	Porosity	Fiber Content
Material type	Refs. [16,17,18,19,20]	Refs. [17,18,20]	Ref. [21]		Ref. [22]		Ref. [17]	Ref. [17]
Printing temperature	Refs. [23,24,25,26]	Refs. [26,27,28,29,30]	Refs. [25,30]		Ref. [30]	Ref. [31]		
Printing speed	Refs. [23,24,26,32]	Refs. [26,28,30,33]	Ref. [4]		Ref. [4]	Ref. [9]		
Layer thickness	Refs. [18,23,24,26,34,35]	Refs. [18,26,27,29,30,33]	Refs. [21,30,36]	Ref. [37]	Refs. [22,30]			Refs. [26,27,35,36,37]
Scanning space	Ref. [23]	Refs. [27,28,33,38]	Ref. [36]					Refs. [27,36]
Stacking orientation	Ref. [18]	Ref. [18]			Ref. [22]			
Fiber orientation	Refs. [17,19,39,40]	Refs. [17,28,41]	Ref. [40]	Ref. [41]			Ref. [17]	Ref. [17]
Fiber content	Refs. [17,39,42]	Refs. [17,41,43]	Ref. [21]	Refs. [41,43]	Ref. [22]		Ref. [17]	

**Table 2:** Parameters of commonly used fibers and resins in CFRTPCs-FDM.

Material Parameters	Reinforcing Fibers			Matrix Resins			
	CF	GF	KF	PLA	ABS	PA	PEEK
Density (g/cm^3^)	1.27–1.76	1.5	1.2	1.25	1.04	1.1	1.32
Tensile Strength (MPa)	700	590	610	15.5–72.2	36–71.6	54	97
Tensile Modulus (GPa)	54	21	27	2.02–3.55	0.1–2.413	0.94	2.8
Tensile Strain at Break (%)	1.5	3.8	2.7	0.5–9.2	3–20	260	
Flexural Strength (MPa)	470	210	190	52–115.1	48–110	32	142
Flexural Modulus (GPa)	51	22	26	2.392–4.93	1.917–2.507	0.84	3.7
Flexural Strain at Break (%)	1.2	1.1	2.1	-	-	-	-
Compressive Strength (MPa)	320	140	97	-	-	-	-
Compressive Modulus (GPa)	54	21	28	-	-	-	-
Compressive Strain at Break (%)	0.7	-	1.5	-	-	-	-

## Data Availability

No new data were created or analyzed in this study. Data sharing is not applicable to this article.
